# Large-scale analysis reveals a functional single-nucleotide polymorphism in the 5′-flanking region of *PRDM16* gene associated with lean body mass

**DOI:** 10.1111/acel.12228

**Published:** 2014-05-23

**Authors:** Tomohiko Urano, Masataka Shiraki, Noriko Sasaki, Yasuyoshi Ouchi, Satoshi Inoue

**Affiliations:** 1 Geriatric Medicine, Graduate School of Medicine, The University of TokyoTokyo, Japan; 2 Anti-Aging Medicine, Graduate School of Medicine, The University of TokyoTokyo, Japan; 3 Research Institute and Practice for Involutional DiseasesNagano, Japan; 4 Research Center for Genomic Medicine, Saitama Medical SchoolSaitama, Japan

**Keywords:** aging, body lean mass, genetics, genome-wide association study, PRDM16, single-nucleotide polymorphism

## Abstract

Genetic factors are important for the development of sarcopenia, a geriatric disorder characterized by low lean body mass. The aim of this study was to search for novel genes that regulate lean body mass in humans. We performed a large-scale search for 250K single-nucleotide polymorphisms (SNPs) associated with bone mineral density (BMD) using SNP arrays in 1081 Japanese postmenopausal women. We focused on an SNP (rs12409277) located in the 5′-flanking region of the *PRDM16* (PRD1-BF-1-RIZ1 homologous domain containing protein 16) gene that showed a significant *P* value in our screening. We demonstrated that PRDM16 gene polymorphisms were significantly associated with total body BMD in 1081 postmenopausal Japanese women. The rs12409277 SNP affected the transcriptional activity of PRDM16. The subjects with one or two minor allele(s) had a higher lean body mass than the subjects with two major alleles. Genetic analyses uncovered the importance of the *PRDM16* gene in the regulation of lean body mass.

## Introduction

Loss of skeletal muscle mass and function is a common disorder in the elderly (Karakelides & Nair, 2005; Cruz-Jentoft *et al*., 2010). It is related to a series of diseases such as sarcopenia, osteoporosis, and frailty. Sarcopenia is a common skeletal muscle disease characterized by low lean body mass of muscle tissue, leading to decreased skeletal strength and increased susceptibility to fracture. Sarcopenia is known to reduce the quality of life of the elderly and has recently become a concern in both developing and developed countries. Measuring lean body mass by dual-energy X-ray absorptiometry (DXA) is a good index for the quantity and quality of skeletal muscles (Hansen *et al*., 1999).

Low lean body mass has a strong genetic component, with heritability ranging over 50% (Nguyen *et al*., 1998; Hsu *et al*., 2005; Keen-Kim *et al*., 2006). However, specific genes underlying the variation in low lean body mass are largely unknown. The identification of novel candidate genes that contribute to low lean body mass susceptibility will impact the diagnosis and treatment for disorders such as sarcopenia. Rapid technological advances have made it feasible to pursue large-scale genome-wide association (GWA) studies (Hirschhorn & Daly, 2005; Wang *et al*., 2010). A large-scale association study is an unbiased approach that involves scanning the entire human genome to identify novel genes or genomic regions with modest effects on complex human diseases and traits. A number of large-scale GWA studies have found novel single-nucleotide polymorphisms (SNPs) associated with complex diseases or traits, including bone mineral density (BMD), fat content, and body mass index (Hirschhorn & Daly, 2005; Wang *et al*., 2010).

We previously performed a large-scale analysis of SNPs in 251 Japanese postmenopausal women using the Affymetrix GeneChip Human Mapping 50K Hind array (first-stage analysis) and in 499 Japanese postmenopausal women (second-stage analysis) to identify common genetic variants associated with BMD (Urano *et al*., 2010, 2012). By analyzing the associations between array SNPs and deviations in BMD determined by DXA, we determined that a common variant in the 3′-flanking region of the *GPR98* gene, rs10514346, is a candidate BMD-related polymorphism. The association of rs10514346 with BMD was replicated in an *in silico* analysis of data from the Framingham Heart Study.

Here, we report a large-scale association study for low lean body mass using Affymetrix 250K SNP arrays in a sample of 269 unrelated postmenopausal Japanese women. The association of an SNP (rs12409277) located in the 5′-flanking region of the *PRDM16* (PRD1-BF-1-RIZ1 homologous domain containing protein 16) gene was further confirmed in another postmenopausal Japanese women population comprised 1081 subjects.

## Results

**Figure 1:**
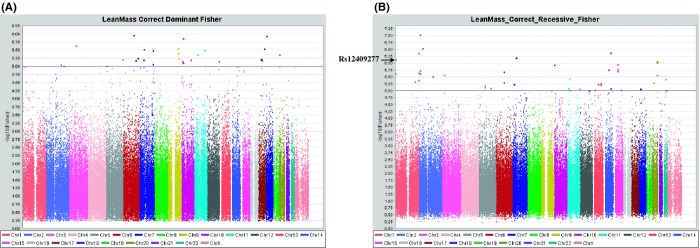
A truncated Manhattan plot showing –log10 (*P* values) for all single-nucleotide polymorphisms (SNPs) associated with body lean mass by 250K SNP array. The gene closest to the SNP with the lowest *P* value at each locus (index SNP) is listed by the dominant (A) and recessive (B) model. The rs12409277 SNP, which is located in the 5′-flanking region of the PRDM16 gene, is associated with lean body mass by the recessive model. Blue and red lines indicate a *P* value of 10^−5^ and 10^−6^, respectively.

**Figure 2:**
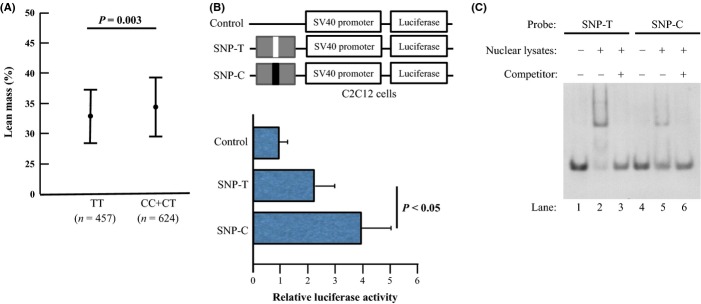
The rs12409277 single-nucleotide polymorphisms (SNPs) affect the lean body mass and PRDM16 transcriptional activity. (A) The percentage of the total body lean mass between the rs12409277 SNP genotypes in the PRDM16 gene. Percentages of the total body lean mass are shown for the TT, CT, and CC genotypes of the rs12409277 SNP. In the analysis, we compare the subjects with one or two minor allele(s) (CT or CC genotype) and the subjects with two major alleles (TT genotype). Scores are expressed as the mean (standard error). The number of subjects is shown in parentheses. The association of the genotype groups with total body lean mass was determined by an unpaired *t*-test. (B) Transcriptional activities affected by rs12409277 SNP, and comparison of allelic variants of rs12409277 SNP analyzed by relative luciferase activity in C2C12 cells and HIT-T15 cells. The values are shown as mean ± SD. Control as pGL3-promoter empty vector. Gray boxes indicate the oligonucleotide unit around the SNPs. The white and black boxes represent the major (SNP-T) and minor (SNP-C) alleles of each SNP, respectively. SV40; simian virus 40. (C) Allele-specific effect of the SNP rs12409277 on the binding of nuclear proteins to the enhancer/promoter region of the human *PRDM16* gene. EMSAs were performed using a ddUTP-labeled T-allele probe or C-allele probe with C2C12 cell nuclear lysates, with or without competition from unlabeled T-allele probe or C-allele probe. Incubation of the C2C12 cell nuclear lysates with the T-allele probe revealed a large increase in intensity of the shifted band (lane 2) as compared to that obtained with the C-allele probe (lane 5). Moreover, the shifted bands were completely abolished by the addition of 125-fold excess unlabeled T-allele or C-allele probes (lanes 3 and 6). Lane 1 and lane 4 were without nuclear lysates (as controls).

We used the Affymetrix 250K SNP GeneChip (262 000 SNPs) to examine the genetic association of SNPs with lean body mass adjusting with age in 269 subjects. The mean (SD) age of the subjects was 64.5 (8.6) years. The basic characteristics of the human subjects are shown in Table 1. For the analysis, we chose 15 662 SNPs with genotype call rates of ≥ 95%, a minor allele frequency (MAF) of ≥ 10%, and a Hardy–Weinberg equilibrium (HWE) of ≥ 0.0001 among 262 000 SNPs. First, we selected the SNPs from the Affymetrix 250K SNP array for a dominant model and a recessive model with *P* values < 10^−6^ in the first screening (Fig. 1). Among the SNPs, we identified the rs12409277 SNP, which is located in the 5′-flanking region of the *PRDM16* gene, as a strong candidate. Previous reports have shown that PRDM16 plays an important role in the differentiation of muscle cells (Sun *et al*., 2011; Richards *et al*., 2012). Thus, we focused on and genotyped the rs12409277 SNP for further analysis in 1081 postmenopausal women. The mean (SD) age of the subjects was 65.1 (9.4) years. The basic characteristics of the human subjects are shown in Table 1. We compared the lean body mass between subjects carrying at least one minor allele and those lacking the minor allele using an unpaired *t*-test. Subjects with one or two minor alleles had significantly higher lean body mass (Fig. 2A, Table 2). We also compared the total body fat mass and bone mineral density between subjects carrying at least one minor allele and those lacking the minor allele (Table 2). There are no significant differences in the fat mass and bone mineral density between the genotypes.

**Table 1:** Basic characteristics in the study

Items	Mean + SD
Number of the subjects	1081
Age (years)	65.1 + 9.4
Body weight (kg)	51.2 + 8.0
Body height (cm)	150.8 + 6.2
BMI (kg m^−2^)	22.5 + 3.1
Fat mass (%)	31.9 + 7.7
Lean mass (%)	34.2 + 3.5
Bone mineral density (g cm^−2^)	0.98 + 0.15

SD, standard deviation.

**Table 2:** Comparison of background data between subjects bearing at least one C allele (CC + CT) and subjects with no C allele (TT) at rs1209277 SNP

Items	Genotype (mean + SE)		*P* value
	TT	CC + CT	
Number of the subjects	457	624	
Age (years)	65.1 + 0.4	65.1 + 0.3	NS
Body weight (kg)	51.0 + 0.3	51.4 + 0.3	NS
Body height (cm)	150.6 + 0.2	150.9 + 0.2	NS
BMI (kg m^−2^)	22.5 + 0.1	22.6 + 0.1	NS
Fat mass (%)	31.8 + 0.4	31.8 + 0.3	NS
Lean mass (%)	33.8 + 0.2	34.5 + 0.1	0.003
Bone mineral density (g cm^−2^)	0.98 + 0.01	0.98 + 0.01	NS

SE, standard error; SNPs, single-nucleotide polymorphisms.

The rs12409277 SNP was located in the 5′-flanking region of the *PRDM16* gene, a region that could putatively affect transcriptional activity. To examine whether rs12409277 SNP would affect transcriptional activity, we performed a luciferase assay using the myoblast-like cell-line C2C12 cells that expressed PRDM16 (Sun *et al*., 2011). Between the major and minor alleles at the locus, the clones containing rs12409277 SNP showed significant differences in transcriptional activity (Fig. 2B). These data suggest that the rs12409277 SNP affects the transcriptional activity of the *PRDM16* gene.

If the T to C change in the SNP rs12409277 affects the transcriptional activity of the *PRDM16* gene, the allelic change may affect the protein–DNA interaction in this region. Thus, we performed electrophoresis mobility shift assays (EMSAs) to determine whether nuclear factors in lysates could bind to oligonucleotide sequences corresponding to genomic sequences containing the T or C alleles of the SNP rs12409277 and also to determine whether the degree of nuclear factor binding differed between the T and C alleles (Fig. 2C). Incubation of nuclear lysates from C2C12 cells with a probe corresponding to the T allele revealed a shifted band (lane 2). Incubation of nuclear lysates from C2C12 cells with a probe corresponding to the C allele showed a reduction in the intensity of the shifted band (lane 5). The shifted bands were completely abolished by the addition of 125-fold excess unlabeled T-allele probe or C-allele probe (lanes 3 and 6). These data suggest that the specific binding of nuclear proteins to the oligonucleotide corresponding to the SNP rs12409277 region is reduced by the T to C change.

## Discussion

Recent technological advances have made it feasible to pursue powerful large-scale association studies (Hirschhorn & Daly, 2005; Wang *et al*., 2010). Large-scale association studies are an unbiased approach that involves scanning the entire human genome to identify novel genes or genome regions with modest effects on complex human diseases or traits. A number of large-scale association studies have revealed novel findings for complex diseases such as obesity, type 2 diabetes, inflammatory bowel disease, and prostate cancer (Hirschhorn & Daly, 2005; Wang *et al*., 2010). Genome-wide association studies of BMD, osteoporosis, and osteoporotic fracture have also been reported (Urano *et al*., 2010, 2012; Richards *et al*., 2012). Recently, GWA studies have also identified common genetic variants associated with lean body mass (Liu *et al*., 2009; Hai *et al*., 2012; Guo *et al*., 2013).

To the best of our knowledge, this is the first large-scale association study for lean body mass variation in the Japanese population. We identified a significant association between the *PRDM16* gene and low lean body mass variation. The findings were further supported by the data from 1081 Japanese postmenopausal women.

We found the rs12409277 SNP affects transcriptional activity of *PRDM16* promoter/enhancer, suggesting that the rs12409277 SNP may regulate the mRNA expression of PRDM16. PRDM16 plays an important role in controlling of differentiation of the brown fat lineage from a progenitor that expresses myoblast markers (Seale *et al*., 2008; Yin *et al*., 2013). These studies have shown that PRDM16 expression promotes brown adipose differentiation and inhibits skeletal muscle differentiation from satellite cells (Seale *et al*., 2008; Yin *et al*., 2013). A previous report has shown that PRDM16 was highly expressed not only in interscapular brown adipose tissue, but also in subcutaneous white adipose tissue (Seale *et al*., 2011). This report also has shown that *aP2-Prdm16* transgenic mice have significantly increased lean mass. Together with these reports, our data suggest that the amount of PRDM16 may regulate not only brown adipose metabolism but also white adipose and muscle cell metabolism.

Lean body mass (%) is influenced not only by actual lean body mass but also by fat mass. For example, in individuals who are overweight or obese, lean body mass (%) should be low even if absolute muscle mass (g) has not decreased. Moreover, given the fact that PRDM16 expression promotes brown adipose differentiation, it would reduce fat mass. This, in turn, could increase lean body mass (%) even without an actual change in absolute lean body mass (g). In the analysis, we found a significant association between the *PRDM16* gene and lean body mass but not fat mass. Here, we also have shown that the enhancer activity of the minor C allele of the SNP rs12409277, which produced a phenotype with higher lean mass, was higher than that of the minor T allele. These data suggest that *PRDM16* gene variation may affect the amount of actual lean body mass. During high-fat feeding, aP2-Prdm16 mice gained significantly less weight than their age- and sex-matched WT littermates. These data suggest that altering the expression of PRDM16 may affect both the differentiation of the myoblasts and the muscle weight in mammals.

After demonstrating differing levels of transcriptional activities between the two alleles of the SNP rs12409277, we sought to identify the nuclear factors involved. EMSAs showed that nuclear factors interacted with probes containing the *PRDM16* enhancer sequence with both the T and C alleles of the SNP rs12409277. Both the T- and C-allele probes detected a gel shift band, but there was an increase in intensity of the upper band with the T-allele probe as compared to the C-allele probe. Although both the luciferase assays and EMSA show a difference between the T and C alleles of the SNP rs12409277, we could not identify a transcription factor that differentially bound to the two alleles and thus would account for the different transcriptional activities of the human *PRDM16* enhancer/promoter associated with the T and C alleles. We propose a model in which a transcriptional factor represses *PRDM16* transcriptional activity by binding to the T allele but not the C allele. It is likely that the T allele plays a more significant role in repressing transcriptional activity, potentially explaining the significantly stronger transcriptional activity of the C allele. In the future study, it will be important to identify the transcriptional repressor that binds the human *PRDM16* enhancer/promoter region.

Previously, the association of metabolic syndrome with a 500K and a 50K SNP gene chip was investigated in the Framingham Heart Study (Park *et al*., 2009). These association tests have shown that SNPs within the *PRDM16* gene were associated with metabolic syndrome phenotypes (Park *et al*., 2009). Recently, a GWA study identified the SNPs in the *PRDM16* gene as a susceptibility loci for the common migraine in the general population (Chasman *et al*., 2011). Moreover, a GWA study identified five loci, including the SNPs in the *PRDM16* gene, influencing facial morphology in the European population (Liu *et al*., 2012). Metabolic syndrome, common migraine, and facial morphology were related to muscle metabolism by SNPs in the *PRDM16* gene. Thus, it is possible that variation in the *PRDM16* gene influences muscle-associated diseases. Future studies are required to demonstrate the association between the SNPs near and in the *PRDM16* gene and the other diseases, especially those related to muscle quality and quantity.

The present study has a limitation in that it included only Japanese postmenopausal women. Whether our findings remain true for men, premenopausal women, or other races is unknown. Future studies are required to analyze the association between this rs12409277 functional SNP and lean mass in other populations.

In summary, our large-scale association study, in conjunction with the known functional involvement of PRDM16 in muscle metabolism, suggests that polymorphisms in the 5′-flanking region of the PRDM16 gene significantly contribute to low lean body mass variation. The mechanisms underlying the observed associations merit further investigation. Taken together, our results suggest that the PRDM16-related signaling pathway could be critical in the regulation of body lean mass. In conclusion, we have shown an association between the rs12409277 functional SNP in the *PRDM16* gene and BMD in Japanese postmenopausal women. Therefore, PRDM16 genotyping may be beneficial in the prevention and management of sarcopenia. The present findings regarding the correlation of PRDM16 polymorphism with lean body mass provide a promising new direction for the clinical management of sarcopenia that could lead to the development of new diagnostic markers as well as therapeutic options based on this molecular target.

## Experimental procedure

### Large-scale association study

Dual-energy X-ray absorptiometry (DXA) scans of the total body were performed to determine lean body mass, fat mass, and bone mineral density (DPX-L machine; GE Medical Systems Lunar Corporation, Madison, WI, USA). In the first screening, we performed a large-scale association to select SNPs based on *P* values using 269 Japanese postmenopausal women. The basic characteristics of the human subjects are shown in Table S1 (Supporting information). We used the Affymetrix 250K SNP GeneChip (262 000 SNPs) to examine the genetic association of SNPs with lean body mass according to the manufacturer’s protocol. In brief, we selected autosomal SNPs with genotype call rates of ≥ 95%, a minor allele frequency (MAF) of ≥ 10%, and a Hardy–Weinberg equilibrium (HWE) of ≥ 0.0001. We analyzed the association between lean body mass and SNP under the assumption of the dominant and recessive models for a minor allele in each SNP, using the quantitative trait loci (QTL) estimation model, as previously described (Urano *et al*., 2010, 2012).

For each SNP in the QTL analysis, the genotypic value (lean body mass) was divided into two parts: the set of genotypic values corresponding to individuals with one or two minor allele(s) vs. with two major alleles with one or two major allele(s) vs. with two minor alleles. The difference between these two sets was evaluated by the threshold model based on the receiver operating characteristic (ROC) curve, considering the sensitivity (true positive) and specificity (false negative) rates. The ROC curve shows the trade-off between sensitivity and specificity, and it is used to evaluate a diagnostic test. To evaluate the effects of the genotypes on the lean body mass, the lean body masses were categorized into two groups: scores that are lower than and those that are higher than the threshold value of the lean body mass. We estimated the threshold of the lean body mass for each SNP to provide the lowest *P* value among all of the simulated thresholds. The association between lean body mass and SNP was evaluated by calculating the relative risk and its 95% confidence interval, and performing Fisher’s exact test. We selected the SNPs from the Affymetrix 250K SNP array for a dominant model and a recessive model with lower *P* values (*P* < 10^−6^) in the screening. Among the SNPs, we identified the rs12409277 SNP, which is located in the 5′-flanking region of the *PRDM16* gene, as a strong candidate. Previous reports have shown that PRDM16 plays a pivotal role in the differentiation of muscle cells (Sun *et al*., 2011; Richards *et al*., 2012). Next, the associations between lean body mass and rs12409277 SNP were analyzed using the data from 1081 postmenopausal women (Table 1). The rs12409277 SNP, which is located in the 5′-flanking region of the PRDM16 gene, was genotyped using the TaqMan PCR method with Assays-on-Demand SNP Genotyping Products and protocols (Applied Biosystems, Foster City, CA, USA). To evaluate the impact of the genotypes on lean body mass, we compared the lean body mass of the groups of subjects classified according to rs12409277 SNP genotypes using an unpaired *t*-test. In the analysis, we compare the subjects with one or two minor allele(s) (CT or CC genotype) and the subjects with two major alleles (TT genotype).

### Cell culture

C2C12 cells were purchased from the American Type Culture Collection (Manassas, VA, USA). Cells were cultured in advanced DMEM (Invitrogen, Carlsbad, CA, USA) with 10% fetal bovine serum, 100 U mL^−1^ penicillin, and 100 μg mL^−1^ streptomycin.

### Luciferase assay

We synthesized double-stranded oligonucleotides containing either a single copy or four concatenated copies of either the major or minor allele for a 19-bp region centered on rs12409277 SNP. We constructed luciferase reporter plasmids by cloning the oligonucleotides into the pGL3-promoter vector (Promega, Madison, WI, USA) upstream of the simian virus 40 promoter. pGL3-promoter vectors containing oligonucleotides were transfected into C2C12 cells together with the phRL-TK vector (Promega), an internal control for transfection efficiency, using FuGENE HD Transfection Reagent (Promega). After 48 h, we collected the cells and measured luciferase activity with the dual-luciferase reporter assay system (Promega).

### Statistical analysis

Data from the samples are expressed as the mean + standard error. The differences between the mean values were analyzed using an unpaired Student’s *t*-test.

#### EMSA

Nuclear extracts were prepared from C2C12 cells following standard procedures. The dsDNA probes containing the SNP rs12409277 were rs12409277-T, 5′-TTTCTGTCCT TTAAAAGCTCTGATGAAATTTTGATTGAAGT-3′ and rs12409277-C, 5′- TTTCTGTCCTTTAAAAGCTCCGATGAAATTTTGATTGAAGT-3′.

Probes were labeled with ddUTP. Each gel shift reaction (20 μL) included 0.8 ng DIG-labeled probe, 10 μg nuclear extract, 1 μg poly [d(I-C)], and 0.1 μg poly l-lysine and was incubated for 15 min at room temperature. For competition experiments, additional unlabeled dsDNA probes were used. A 125-fold excess of unlabeled probe was pre-incubated with nuclear extracts for 20 min before the addition of the labeled probe. EMSAs were performed according to the instructions for the DIG Gel Shift Kit, 2nd generation (Roche, Mannheim, Germany).

## Supporting Information

**Table S1** Basic characteristics in the large-scale analysis.
