# Supplementary material for: Large-scale analysis reveals a functional single-nucleotide polymorphism in the 5′-flanking region of PRDM16 gene associated with lean body mass
Source: Aging Cell. 2014 May 23;13(4):739–43. doi: 10.1111/acel.12228 (PMC4326941; doi:10.1111/acel.12228)
Supplement: Supplementary file 1 — Table S1 Basic characteristics in the large-scale analysis. [file acel0013-0739-sd1.doc]

**Supplementary Table S1** Basic characteristics in the large-scale analysis.

Items Mean + SD

Number of the subjects 269

Age (years) 64.5 + 8.6

Body weight (kg) 51.8 + 7.8

Body height (cm) 151.7 + 5.6

BMI (kg/m2) 22.5 + 3.3

Fat Mass (%) 31.8 + 7.8

Lean mass (%) 34.5 + 3.4
